# Radiomic detection of microscopic tumorous lesions in small animal liver SPECT imaging

**DOI:** 10.1186/s13550-019-0532-7

**Published:** 2019-07-25

**Authors:** Dániel S. Veres, Domokos Máthé, Nikolett Hegedűs, Ildikó Horváth, Fanni J. Kiss, Gabriella Taba, Edit Tóth-Bodrogi, Tibor Kovács, Krisztián Szigeti

**Affiliations:** 1 Department of Biophysics and Radiation Biology, Semmelweis University, Budapest, H-1094 Hungary; 2 CROmed Translational Research Centers Ltd, Budapest, H-1047 Hungary; 3 Dosimetry and Radioprotection Service, Semmelweis University, Budapest, H-1082 Hungary; 4 Institute of Radiochemistry and Radioecology, University of Pannonia, Veszprém, H-8200 Hungary

**Keywords:** Radiomics, Liver cancer, Early detection, SPECT, Quantification, SUV, Skewness, Kurtosis

## Abstract

**Background:**

Our aim was to present a new data analysis technique for the early detection of tumorous lesions using single-photon emission computed tomography (SPECT) imaging. Beyond standardized uptake value (SUV) and standardized uptake concentration (SUC), the skewness and kurtosis parameters of whole liver activity distribution histograms were examined in SPECT images to reveal the presence of tumorous cells.

**Methods:**

Four groups of mice were used in our experiment: a healthy control group, a group of *obese* mice with high body mass index, and two tumorous groups (*primary liver cancer group* with chemically induced hepatocellular carcinoma (HCC); *metastatic liver tumor group*—xenograft of human melanoma (HM)). For the SPECT measurements, ^99m^Tc-labeled aggregated albumin nanoparticles were administered intravenously 2 h before the liver SPECT scans (NanoSPECT/CT, Silver Upgrade, Mediso Ltd., Hungary) to image liver macrophages. Finally, SUV, SUC, skewness, and kurtosis of activity distributions were calculated from segmented whole liver volumes.

**Results:**

HCC animals showed moderate ^99m^Tc-albumin particle uptake with some visually identified cold spots indicating the presence of tumors. The visual detection of cold spots however was not a reliable marker of tumorous tissue in the metastatic group. The calculated SUV, SUC, and kurtosis parameters were not able to differentiate between the healthy and the tumorous groups. However, healthy and tumorous groups could be distinguished by comparing the skewness of the activity distribution.

**Conclusion:**

Based on our results, ^99m^Tc-albumin nanoparticle injection followed by liver SPECT activity distribution skewness calculation is a suitable image analysis tool. This makes possible to effectively and quantitatively investigate liver macrophage inhomogeneity and identify invisible but present liver cold spot lesions. Skewness as a direct image-derived parameter is able to show altered tissue function even before the visual manifestation of liver tumor foci. The skewness of activity distribution might be related to an inhomogeneous distribution of macrophage cells as a consequence of microscopic tumor burden in the liver.

**Electronic supplementary material:**

The online version of this article (10.1186/s13550-019-0532-7) contains supplementary material, which is available to authorized users.

## Introduction

The liver is one of the most affected organs for metastatic disease because of its dense vascular network. It is supplied with oxygenated blood via the hepatic artery (25%) and the portal vein (75%) [1]. Liver metastasis (as secondary tumor) is a malignant alteration of the organ. Metastatic liver tumors occur 20 times more frequently than primary liver tumors. However, at an early well-treatable stage, liver metastasis may not show any specific symptoms. The symptoms often appear only in later stages when the tumorous cells have already infiltrated parts of the liver too large for curative intent therapy. Consequently, the survival of liver metastatic patients varies only between several months and a few years depending on the type of the primary cancer, the size and number of metastases, and the applicable treatment based on disease stage. Thus, early and precise diagnosis of liver metastasis is a critical and important step of the full diagnostic protocol for therapy plans.

Ultrasound (US) and computed tomography (CT) are the most widely used imaging techniques for liver metastasis. However, these modalities are not able to measure or detect altered functional features of the liver. CT and US detect only the presence of tumorous cells with acceptable sensitivity in a size range above 10 mm. In these cases, the liver function has already been affected and multiple small metastases could be present, too. Further quantitative and functional diagnostics of malignant alterations include positron emission tomography (PET) [2, 3] and dynamic contrast-enhanced magnetic resonance imaging (MRI) [4] with gadoxetic acid [5] or gadoxetate disodium [6, 7]. Several radiotracers have been investigated for single-photon emission computed tomography (SPECT) that is able to measure the liver function in different ways [2, 3, 5, 8, 9].

Nowadays, there is an increased interest in radiomics. Radiomics can extract additional quantitative data from functional PET and SPECT scans beyond radioactive concentration based data. Standardized uptake value (SUV) is the most widely used clinical PET parameter. SUV could be calculated in different ways [10–12]. Other quantitative metrics such as metabolic tumor volume (MTV) and total lesion glycolysis (TLG; SUV_mean_ × MTV) are reported to yield better discriminatory, predictive, and prognostic information compared to SUV [13–19]. All these calculated PET metrics have their counterparts in SPECT measurements. The functional liver volume (FLV) represents the volumetric measure of liver function, liver to spleen ratio (L/S_mean_) represents the global magnitude of liver function, and total liver function (TLF) as the most suitable imaging parameter of liver function in clinics represents the integral liver function. FLV, L/S_mean_, and TLF are quantitated from organic anion transporter substrate-based SPECT measurements [18].

Unfortunately, the reliability and correctness of the abovementioned quantitative imaging parameters are highly variable. In general, these metrics are all affected by the obesity of the patient and the weight and volume of the organ. Thus, it is currently unknown which imaging metrics are the closest to real clinical liver functions and patient outcome [18].

In a radiomics approach, one characterizes the heterogeneity (e.g., intra-tumoral differences in molecular and cellular characteristics, necrosis, fibrosis, metabolism, hypoxia, and angiogenesis) of the tumors by different quantitative metrics usually called textural parameters [20]. These metrics could improve tumor phenotypic characterization, prediction, and prognosis of the tumor and could eventually lead to personalized treatment [21–26].

The primary objective of our study was to discover the presence of tumorous burden as early as possible—even before it is directly visible in liver SPECT images. We exploited the fact that tumorous lesions create inhomogeneity in the distribution of macrophages inside the liver. Although there are several different SPECT image textural parameters investigated in the literature, most of them are not straightforward in their biological representation or time-intensive to determine. From literature reports, two simple radiomics parameters stand out: determination of kurtosis and skewness is very common in different imaging modalities and many image quantitation methods [24–26]. We previously suggested an imaging method based on the distribution of macrophages (mainly Kupffer cells) that could show tumorous lesions in the liver [27].

As our study aims to bring a clinically translatable and simple method to the forefront, we wished to concentrate on the assessment of these easily calculated parameters. The use of the skewness and kurtosis of a VOI histogram can avoid the need for more complex, artificial intelligence-based calculations. By resorting to the simply calculated statistical parameters skewness and kurtosis of the activity distribution in the whole liver, our aim was to estimate and characterize the presence of invisible tumorous burden in the organ in a well-described mouse liver metastasis model. Our aim was to bridge the gap between quantitative imaging heterogeneity parameters and functional liver imaging.

## Methods

The aim of this study was to assess different imaging and radiomics parameters for the distinction between microscopic liver tumor lesions and healthy liver volumes using ^99m^Tc-labeled protein nanoparticle imaging. For the realistic modeling of clinical problems, we used a “healthy” population of mice that consisted of two groups, “control” and “obese.” A second, “tumorous” population of mice was included representing one “metastatic” and one “primary” liver tumor group.

### Animals

Ethical permission was obtained from the institutional and national boards (see the “Ethics approval and consent to participate” section in the Declarations). Animals were fed ad libitum and maintained under controlled temperature, humidity, and light conditions.

Four groups of mice were examined in our experiment, a *healthy* population with two groups and a *tumorous* population with two other groups of mice. The first healthy group (*control*) contained different strains of mice (BALB/c, C3H, C57BL/6) to mimic population inhomogeneity with increased external validity (*n* = 2/breed, total *n* = 6, 32.4 ± 10.6 g, (mean ± SD)). Mice with higher body mass index—APP/PSEN1 transgenic mice and a C3H mouse strain with high body mass—(*n* = 8, 46.9 ± 14.5 g (mean ± SD) were used to model healthy but obese cases (*obese* group). Cancer-prone matrilin-2 knocked-out (KO) transgenic (MATN2) mice (*n* = 9, 38.5 ± 1.9 g (mean ± SD)) with chemically induced hepatocellular carcinoma were used as the *primary liver cancer* group. The fourth group was the *liver metastatic* group where severe combined immunodeficiency mice (*n* = 4; 21.3 ± 0.9 g (mean ± SD)) were inoculated with human melanoma cells (HT-168-M cell line, Avidin Ltd., Hungary) in the spleen. This model leads to fast liver metastasis formation. Mice were sourced from Innovo Ltd., Hungary.

### Induction of tumorous models

The *primary* cancer group was induced by intraperitoneal diethylnitrosamine (Sigma-Aldrich, St. Louis, USA) injection in 15-day-old mice. The lack of matrillin-2 can increase the tumor proliferation in the liver [28]. The mice were investigated 4 months after the induction when liver tumors have reached the advanced multifocal status.

The *metastatic* group was induced by intrasplenic injection of a human melanoma HT168-M cell line (0.1 mL, ~ 5 × 10^5^cells). This xenograft has high liver-colonizing capacity [29]. These mice were imaged only two months after the cell inoculation to mimic the early stage and the small size of metastatic lesions.

### In vivo SPECT imaging protocol

Mice were anesthetized with isoflurane (3.5–4% induction and then reduced to 1.5–2% for maintenance of anesthesia) for the radiopharmaceutical administration and later again for imaging. Two hours before SPECT imaging, 87.2 ± 8.3 MBq (*injected activity*; mean ± SD) of ^99m^Tc-protein nanoparticles, reconstituted from a clinically marketed human serum albumin-based kit for radiopharmaceuticals (Nano-Albumon® particles with < 200 nm size; Medi-Radiopharma Ltd., Budapest, Hungary; 2–5 GBq/mg specific activity), were administered intravenously. Animals were weighed prior to imaging.

SPECT (NanoSPECT/CT Silver Upgrade, Mediso Ltd., Budapest, Hungary) measurements were performed with multi-pinhole mouse collimators (pinhole diameter 0.7 mm). Abdominal SPECT scans were performed with 17–25 min scan times. The detection peak energy for ^99m^Tc was set to 140 keV with a 20% wide symmetric energy window. The reconstruction of the raw SPECT data was performed with 0.33 mm isovoxel size while the field of view was centered to the abdominal region. All reconstructions were made by the instrument’s dedicated HiSPECT (Scivis GmbH, Göttingen, Germany) software. The reconstruction algorithm and settings may have an effect on the skewness and kurtosis values of voxel radioactivity distribution histograms. To minimize this source of error, we always applied the same reconstruction settings (“high” smoothing (33% Gauss), “high” reconstruction voxel size (0.33 μm), “low” iteration number (3 iterations) of the software settings). (In the *primary* group, we used the same raw data that were published before [27] for further evaluations.)

### Histology

After imaging the animals were euthanized by pentobarbital sodium injection (400 mg/ml Euthasol, Medicus Partner Kft., Hungary). Autopsy was performed immediately and the liver was removed and stored in 4% formaldehyde-PBS solution. Afterwards, the presence of tumorous lesions was examined by histology in Hematoxylin-Eosin stained 10 μm thin sections.

### Image and data analysis

After the reconstruction of image volumes, segmentation of the whole liver was made using the VivoQuant software (version 1.22., inviCRO, Boston, US) by two independent observers. At first, the liver region was visually cropped and an Otsu thresholding method was applied to obtain the reliable and automatic delineation of liver versus non-liver volumes [30]. The possible remnants of the spleen volume were then eliminated manually to get the final volume of interest (VOI).

These VOIs were compared to each other while two naïve observers were looking for liver cold spots. Four different image parameters (SUV, SUC, skewness, and kurtosis) were calculated from the segmented livers using MATLAB software (version 7.10.0., MathWorks, US) (the script and the calculated parameters are available in the Additional file 1) based on the following equations:

1 $$ \mathrm{SUV}=\frac{A_{\mathrm{liver}}\bullet m}{A_{\mathrm{total}}\bullet {V}_{\mathrm{liver}}}, $$

2 $$ SUC=\frac{A_{\mathrm{liver}}}{A_{\mathrm{total}}\bullet {V}_{\mathrm{liver}}}, $$

3 $$ \mathrm{skewness}=\frac{\frac{1}{n}\bullet \sum \limits_{i=1}^n{\left({x}_i-\overline{x}\right)}^3}{{\left(\sqrt{\frac{1}{n-1}\bullet \sum \limits_{i=1}^n{\left({x}_i-\overline{x}\right)}^2}\right)}^3}, $$

4 $$ \mathrm{excess}\ \mathrm{kurtosis}=\frac{\frac{1}{n}\bullet \sum \limits_{i=1}^n{\left({x}_i-\overline{x}\right)}^4}{{\left(\frac{1}{n-1}\bullet \sum \limits_{i=1}^n{\left({x}_i-\overline{x}\right)}^2\right)}^2}-3, $$

where *V*_liver_ is the radioactivity volume in cm^3^, *A*_liver_ is the summarized liver activity in kBq, *A*_total_ is the administered radioactivity in kBq, and *m* is the mass of the animal in grams. In Eqs. 3 and 4. *x*_*i*_ represents the radioactivity in each VOI voxel, *x̅* is the arithmetic mean of *x*_*i*_ values, and *n* is the number of voxels in the VOI.

### Statistical calculations

Before the detailed analysis, the effect of manual segmentation process had to be verified. For this reason, we compared each of the resulting parameters based on two paired segmentations made by two independent observers. At first, difference plots (also known as Bland-Altman plot) were created for each imaging parameter, where the difference versus the mean of the repeated measures were plotted. Monotonic association (trend) between the difference and the mean of the repeated measures was examined with Spearman’s correlation tests. We preferred non-parametric methods due to the assumed non-normality and heteroscedasticity to describe the agreement between the two researchers. Therefore, Kendall *W* and the corresponding *p* values were calculated for each parameter. To check the systemic difference between the researchers a linear correlation was assumed. The slope and the intercept were determined based on this model. Hereinafter, we used the VOIs of the first evaluator.

Box-plots were created to comparing the differences of each parameter among the groups. Mann-Whitney *U* tests were performed to assess the differences of averages between *tumorous* and *healthy* populations. Multiplicity correction was not applied.

To judge the performance of the parameters’ classification between *tumorous* and *healthy* animal populations, empirical nonparametric receiver operating characteristics curves (*ROC*) were created. For plotting the *ROC* curve theoretical false and true positive rates (*FPR* and *TPR*) have to be estimated. As a crude estimation we made an assumption on FPR and TPR at each value in our dataset as a threshold for classification. In the case of SUV and SUC we defined cases as positive (assumed to be “tumorous”) if the value was smaller than the threshold. In the case of skewness and kurtosis we defined cases as positive if the value was larger than the threshold. The area under the curve (*AUC*) is estimated by summing the trapezoids enclosed by the points of the ROC curve.

Statistical calculations were made using R (Version 3.2.3. R Core Team, R: A Language and Environment for Statistical, Vienna, Austria, using vegan package for Kendall *W*). Difference plots were made by *R* using BlandAltmanLeh package. Box plots were created by Statistica 64 (Version 13., Dell Inc., Tulsa, US).

## Results

**Fig. 1:**
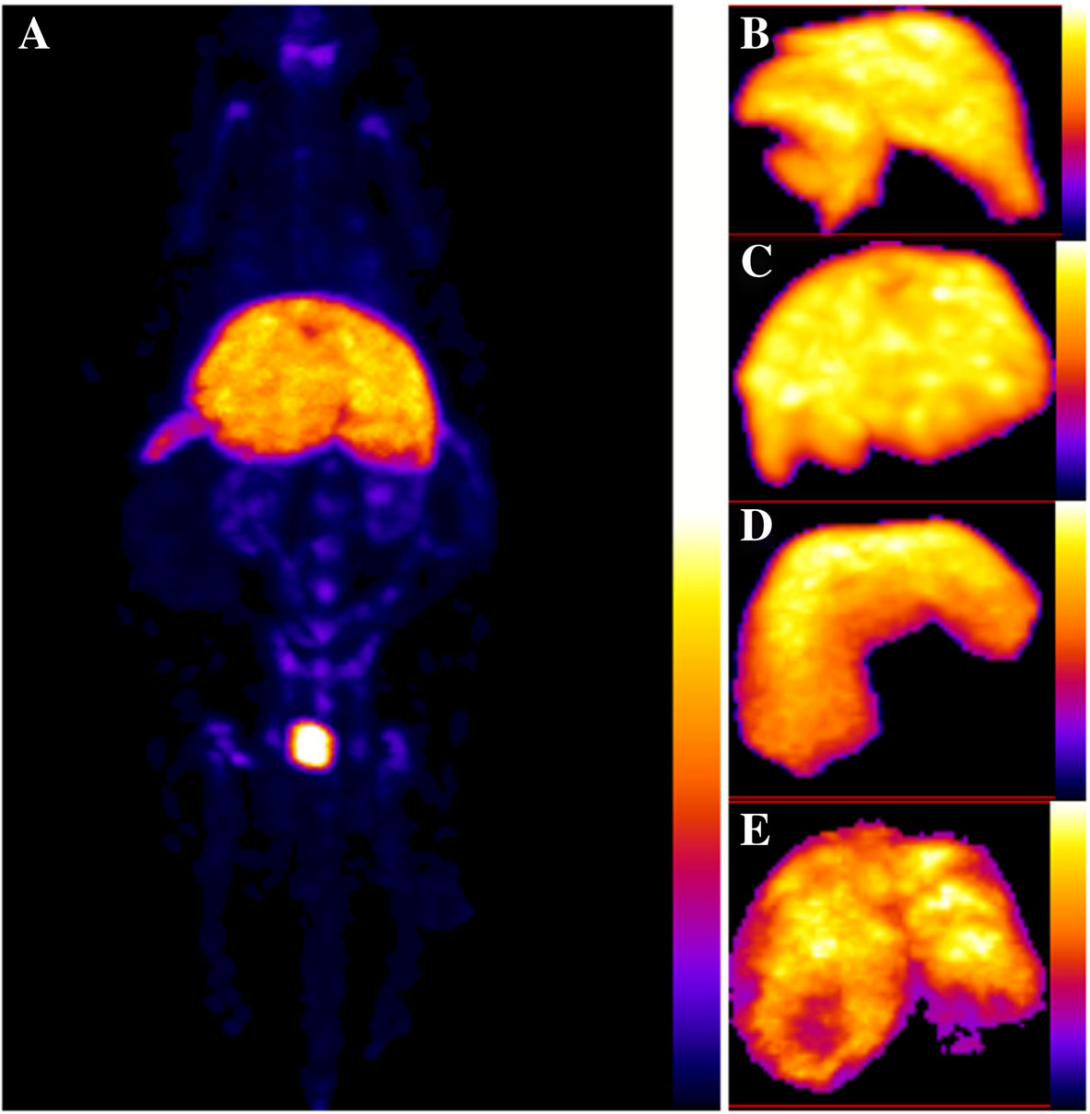
**a** The result of ^99m^Tc-protein nanoparticle whole-body SPECT scan. **b**–**e** Selected projections of the segmented liver in control, obese, metastatic, and primary tumor groups respectively from top to bottom (additional projections are available in the supplementary material; all images are from individual experiments)

The results of whole-body ^99m^Tc-protein nanoparticle SPECT measurements are illustrated in Fig. 1a, while Fig. 1b–e illustrates the segmented livers of *control*, *obese*, *metastatic*, *and primary tumor* mice, respectively.

Cold spots could be visualized in all cases in the *primary liver cancer* group, while 3 of 4 mice showed cold spots or visually increased heterogeneity in the liver in the *metastatic* group.

**Fig. 2:**
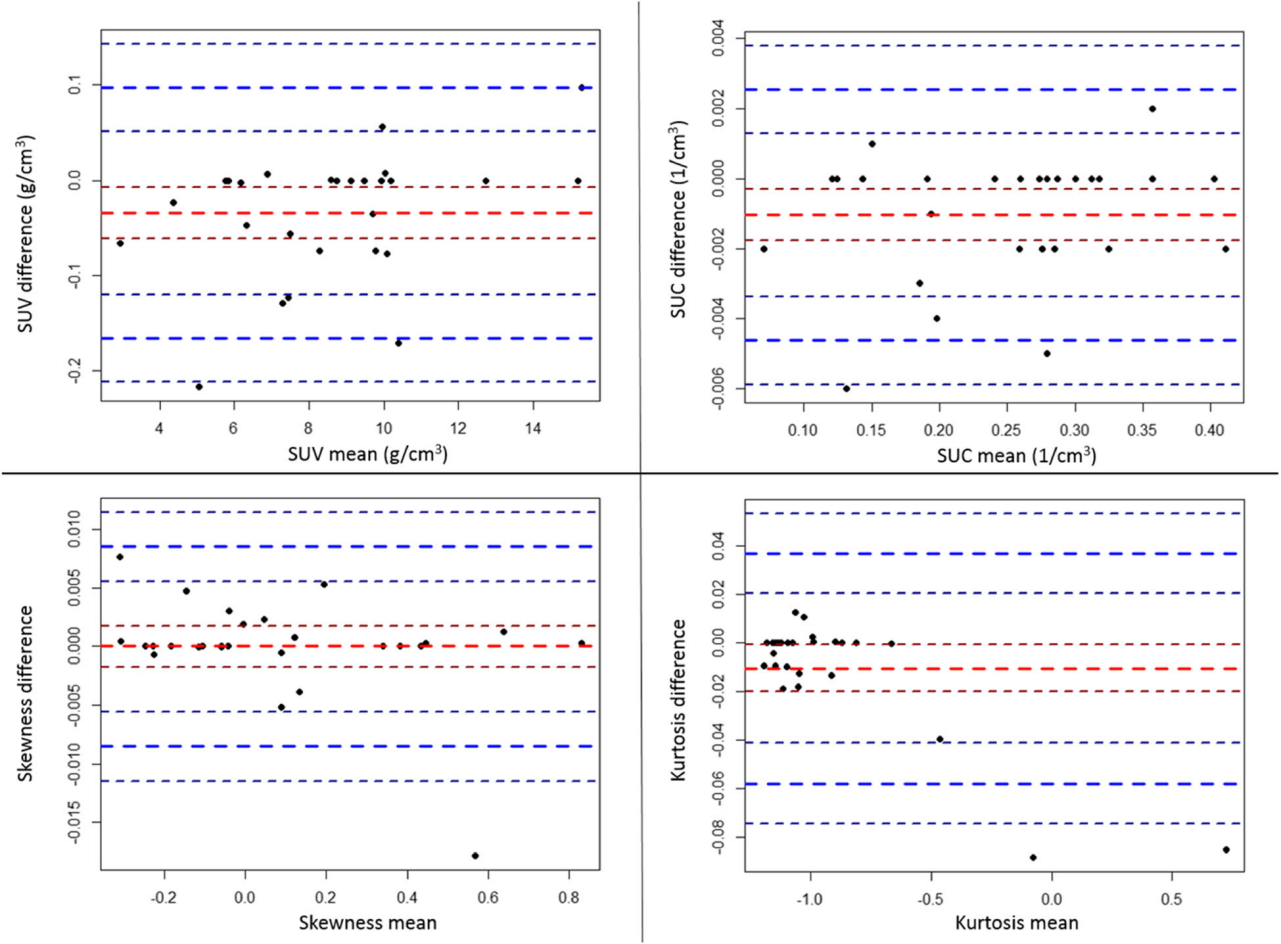
Difference plot for each parameter. Red line: mean of differences (with 95% confidence interval). Blue lines: 95% limits of agreement (with 95% confidence intervals)

Figure 2 illustrates the difference plot for each parameter. In this scatter plot, each point represents an animal. The *Y* value represents the difference of the parameters between the two evaluators, while the *X* value is the mean of the two values. The mean of the differences is represented by a bold red line in the middle (additional red lines represent the 95% confidence interval of the mean). The 95% prediction interval (“limits of agreement”) and its 95% confidence intervals are also plotted in the figure (blue lines). The Spearman correlation test resulted in *p* values of 0.1083, 0.5576, 0.9271, and 0.3596 (SUV, SUC, skewness, kurtosis, respectively). Therefore, we could not reject that there is no trend in the graphs. The mean of the differences is close to 0 at each parameter (Table 1): the differences are 1–2 orders of magnitude smaller than the difference of medians in the *tumorous* and *healthy* populations (Table 2).

**Table 1:** Results of the comparison of two researchers’ segmentations

	Image parameters			
	SUV	SUC	Skewness	Kurtosis
Mean of differences	− 0.0343 (g/cm^3^)	− 0.0010 (1/cm^3^)	< 0.0000	− 0.0105
SD of differences	0.0672 (g/cm^3^)	0.0018 (1/cm^3^)	0.0043	0.0242
Kendall *W*	0.9997	0.9993	0.9994	0.9985
Kendall *p*	< 0.0001	< 0.0001	< 0.0001	< 0.0001
Slope	1.0088	1.0040	0.9950	0.9504
Slope 95% CI	0.9999; 1.0176	0.9966; 1.0124	0.9895; 1.0004	0.9385; 0.9624
Intercept	− 0.1099 (g/cm^3^)	− 0.0020 (1/cm^3^)	0.0004	− 0.0559
Intercept 95% CI	− 0.1906; − 0.0292 (g/cm^3^)	− 0.0042; 0.0002 (1/cm^3^)	− 0.0013; 0.0021	− 0.0679; − 0.0439

*95% CI* 95% confidence interval, *SD* Standard deviation

**Table 2:** Median value of the parameters in each population and group

	Image parameters			
	SUV (g/cm^3^)	SUC (1/cm^3^)	Skewness	Kurtosis
Control (*n* = 6) (healthy+)	11.2130	0.3575	− 0.0522	− 1.1463
Obese (*n* = 8)	9.6105	0.2170	− 0.1647	− 1.1253
Metastatic (*n* = 4)	6.2440	0.2935	0.2351	− 0.8650
Primary (*n* = 9)	7.2130	0.1840	0.3883	− 0.8973
Healthy (*n* = 14)	9.7135	0.2770	− 0.1301	− 1.1359
Tumorous (*n* = 13)	6.3110	0.2580	0.3399	− 0.8973

Table 1 contains the additional numeric results of the comparison of the image parameters calculated by the two researchers. For all image parameters, the Kendall *W* was greater than 0.99 with *p* < 0.0001. Most of the 95% confidence interval of the slope contained 1 and most of the 95% confidence intervals of intercept contained 0. In other cases, the slope and intercept differences were not relevant (it caused < 1% difference).

**Fig. 3:**
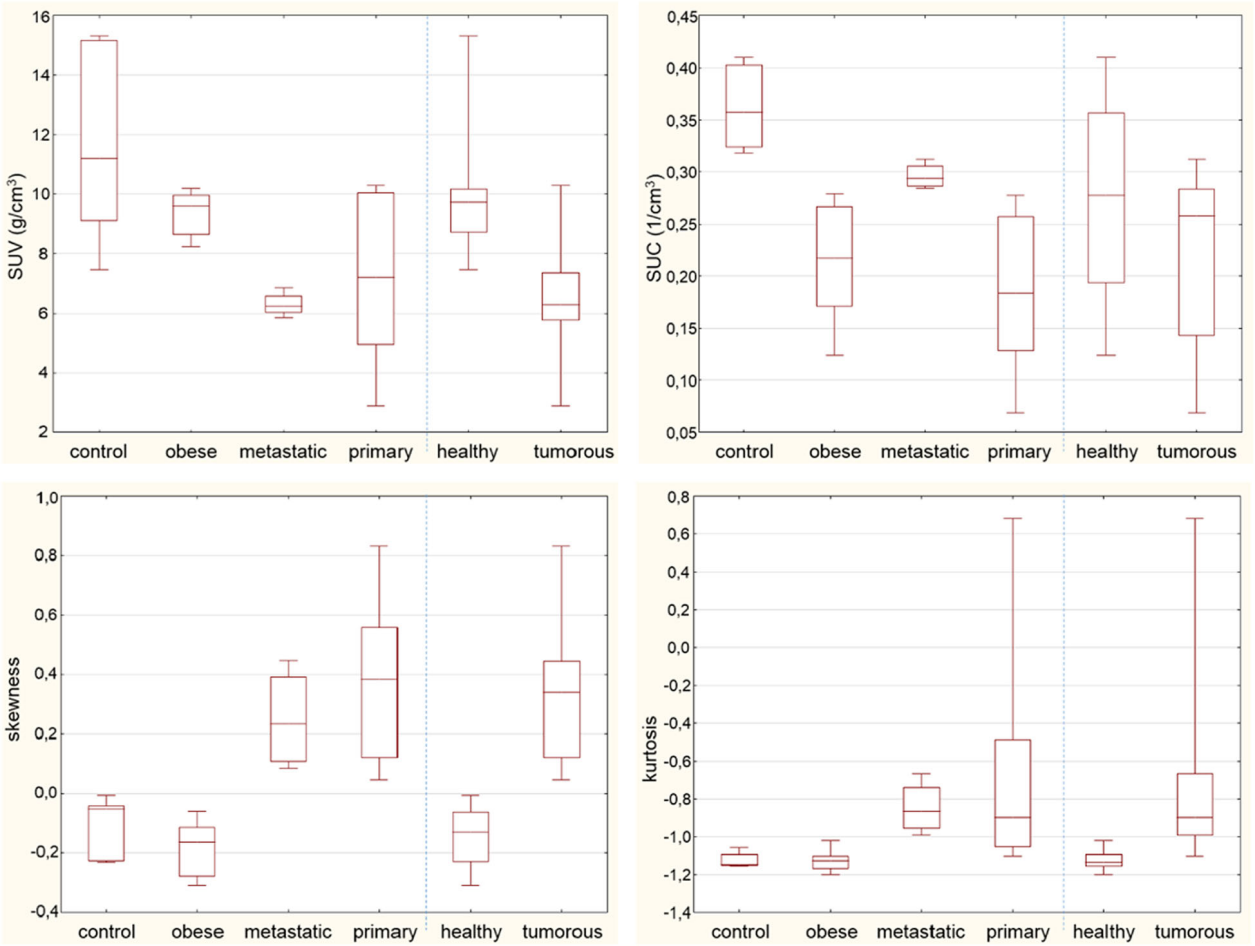
Box plots show the liver volume of interest parameters (SUV, SUC, skewness, and kurtosis) in different groups (control *n* = 6, obese *n* = 8, metastatic *n* = 4, primary *n* = 9, healthy *n* = 14, tumorous *n* = 13) (middle line: median, box: quartiles, whiskers: minimum-maximum)

Subsequently, data of segmentation made by the first observer were used. In Fig. 3, the box plots illustrate the SUV, SUC, skewness, and excess kurtosis values to compare the groups (left side) to each other and to compare *healthy* populations (*control* and *obese* groups together) to *tumorous* populations (*metastatic* and primary groups together) (right side).

*p* values in the Mann-Whitney *U* test were smaller than 5% for SUV (*p* = 0.0036), skewness (*p* < 0.0001), and kurtosis (*p* < 0.0001), but there was no evidence for the difference between medians for SUC (*p* = 0.1093) at this significance level. Table 2 contains the numeric values of the parameters in each population and group.

**Fig. 4:**
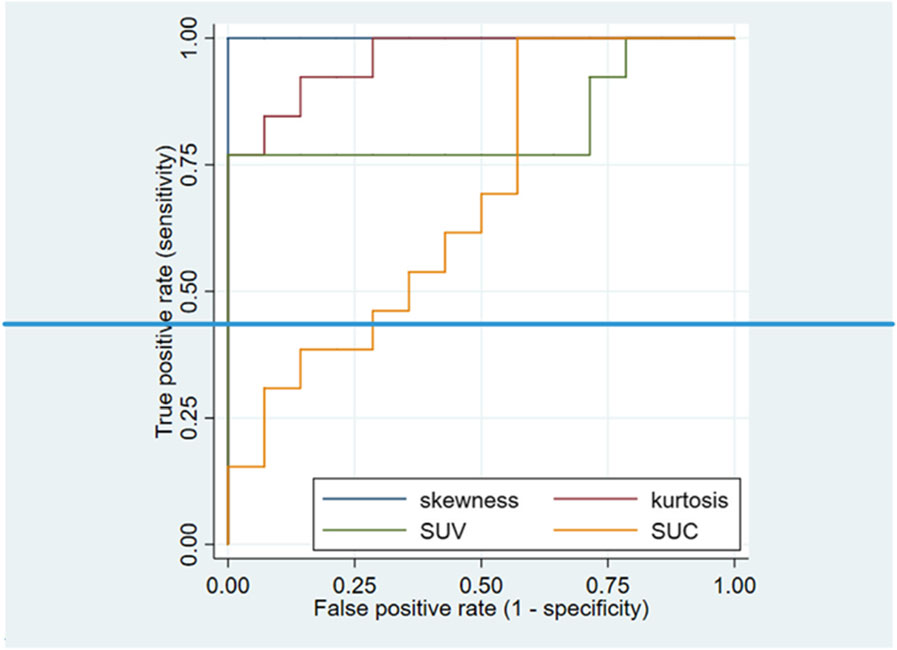
The empirical ROC curve of the four investigated imaging parameters

Empirical ROC curves were produced (Fig. 4) to evaluate the classification of tumorous status based on the above-discussed image parameters. The points in the ROC curve represent sensitivity as the false-positive rate pairs at a given parameter value. The AUC (area under the curve) values were 0.82, 0.68, 1.0, and 0.96 for SUV, SUC, skewness, and kurtosis, respectively. The estimation used for the theoretical ROC curves could be valid only if the ratio of “tumorous” and “healthy” subjects does not differ in the estimated population. In other cases, the ROC results have to be handled cautiously.

The autopsy and histology study has confirmed the results of in vivo SPECT scans. Macroscopic tumorous lesions could be identified in the liver in the *primary* cancer group, while the *metastatic* group showed only microscopic lesions. Autopsy revealed systemic inflammation in one *obese* mouse, which animal was further excluded from the study. There was no further evidence for pathological lesions in the *healthy* population.

## Discussion

Historically, one of the first used semi-quantitative measures was SUV. When SUV calculations were established, it was assumed that VOI activity (or activity concentration for SUC) is linearly proportional to body mass and VOI volume. Unfortunately, these assumptions have been biased [31]. Additionally, application of SUV assumes that mass and VOI volume are measured without any error. To avoid this problem, other values of image quantitation across studies need to be found, for example, different textural parameters.

Several textural quantitative parameters (e.g., parameters of image histogram) were defined in order to evaluate the heterogeneity of CT or MRI images in the last decades [32]. These parameters and widespread radiomics methodology make possible the estimation of the success rate of tumor therapy [33]. This approach is applicable for the estimation of liver metastases, too [34].

Until this time, radiomics-based studies have published textural parameters related only to the tumor or to a small region of interest around the tumor. However, our more practically oriented technique makes it possible to detect the tumor burden of entire organs such as the liver. With this quantitative data analysis method, the skewness and kurtosis of liver radioactivity distribution calculations have proven to be feasible in small animal whole liver SPECT images not just in predefined smaller tumor volumes.

Our study was limited only to the investigation of skewness and kurtosis of liver VOI histograms due to these parameters’ lack of dependence on the total injected activity. Thus, the proposed radiomics methodology remains quantitative without any need for establishing the absolute value of the radioactivity and helps to avoid standardization problems. In addition, skewness and kurtosis are sensitive to the variability (heterogeneity) and extremes of the activity distribution within the VOIs, which variability is caused by increased heterogeneity of macrophage cell distribution and tumorous micro-lesions of the liver [27].

As a result of skewness and kurtosis analysis, cold spots could be described in the liver in the different *tumorous* groups, while no visible liver lesions were detected in the case of *healthy* groups. Based on these outcomes, our method seems to be an appropriate technique that facilitates the quantitative evaluation of altered liver tissue.

The difficulty of creating accurate VOIs and the resulting erroneous VOI results would have been the most important limitations of this technique. Therefore, a comparison of different segmentation processes by two evaluators was required. It was assumed based on the difference plots that there were no relevant differences between these two evaluators. The high (close to 1) Kendall *W* values (with low *p* values) represented great concordance, while the slope and intercept values did not show any relevant systemic difference between the observers. These results have been confirmed by the used segmentation procedure, which was not dependent on the evaluator. The same results were obtained in the case of independent segmentation as well.

Although SUV values were statistically different from each other (according to the Mann-Whitney *U* test result) in *tumorous* and *healthy* groups, there was an overlap between the *healthy* and *tumorous* populations’ SUV values as it can be seen in Fig. 3. This is probably due to the different mass of the animals and the liver VOI activities in the case of tumorous mice.

SUC values were also found to be statistically significantly different and were more predictive in the differentiation between *control* and *tumorous* groups. However, SUC was not an appropriate differentiating parameter in the case of the *obese* group. This could have been expected, because SUC is not proportional to the mass of the subject. These limitations could also be seen on the empirical ROC curves. It is worth noting that in this pilot study, the ROC curves have to be handled cautiously not only because of the small sample size but also because the distribution of the animals was not the same as that of a “general” mouse population. This was due to an intentional overrepresentation of obese animals and animals with small liver metastases.

The highest difference (good but not perfect differentiation and classification) between the two examined evaluators could be observed with the examination of kurtosis.

Altogether, skewness was the best parameter for the classification of the four distinct groups. Based on the empirical ROC curve, the skewness yielded an ideal classification between the *tumorous* and *healthy* groups and well as between the pooled animals from both tumor-bearing groups versus the pooled animals from both healthy groups. In the case of skewness, we recall that this value relates to the voxel histogram distributions and, more specifically, to the third central moment of a given distribution. This moment is in turn dependent on the values at the sides (“tails”) of a histogram. Tumorous animals will have an overall higher number of voxels with less activity in the liver. Thus, the decreased number of voxels with higher activity, leading to a decreased weight on the right side of histograms, will create the remaining activity-avid voxel population being “outliers” on the right side. The skewness calculation yields positive values if there is a pronounced “outlier” population value in the right side (i.e., a prominent “right tail”). Tumorous animals have positive skewness in spite of the bulk of the voxels in their livers being on the left side (containing less activity) because of the effect of this outlier right side group of voxels affecting the third central moment of the histogram, hence determining its skewness towards the positive value.

If we consider the similarity of ratios of specific SPECT volumetric resolutions vs. liver volumes in mouse and in man, we arrive to an easy translation for human liver SPECT volume analysis with our method. The human clinical SPECT voxel volumes however are circa 1000 times larger than the mouse voxels. This also means in an approximation that circa 1000 times more Kupffer cells will be present in one clinical SPECT voxel than in one mouse micro-SPECT voxel. In turn, this increases the hypothetical chances of clinical liver tumor detection sensitivity of our skewness method due to more robust data availability. The main sources of signal in this type of imaging are Kupffer cells. Therefore, a clinical application study of skewness as liver tumor diagnostics is standing a fair chance of being successful, as the signal-to-noise ratio will probably be increased in human livers. On the other hand, skewness depends on the proportion of “cold,” i.e., tumor-affected liver voxels to normal liver voxels and might only change significantly in humans when a large liver volume has already been affected. Thus, it might also well occur that a clinical skewness study would be valuable in patients with advanced liver disease. Before clinical studies, the minimally detectable tumorous voxel fraction therefore should be modeled to achieve acceptable sensitivity.

In our study, properly sized and ^99m^Tc-labeled protein nanoparticles were intravenously administered to mice. We applied a clinically available nanoparticle-based radiopharmaceutical kit that is currently labeled for human bone marrow and inflammation scans. However, the clinical application of this marketed, authorized radiopharmaceutical for the “off-label” use in liver imaging can be straightforward in man. These particles differ from other portal shunt detection particles, which are used for gamma camera imaging [18, 27].

## Conclusion

Based on our small animal ^99m^Tc-albumin nanoparticle SPECT tumorous liver study results, we suggest the use of skewness of liver activity distribution histogram to characterize the inhomogeneity of the whole organ due to tumor infiltration. Invisible tumorous invasion with changed macrophage distribution in the whole liver could be detected and quantified using radiolabeled protein nanoparticle radioactivity distribution skewness. We propose this method of using nanosized protein particles as a new, clinically translatable imaging biomarker of liver tumor burden. The clinical studies benefitting from this translation could be aimed at hidden liver tumor burden estimation and progressive disease as an endpoint, with, e.g., patients with possible liver metastatic involvement from other primary cancers.

## Additional file

Additional file 1 The supplementary file contains the MATLAB script for calculating the parameters, additional example projections in each animal of the segmented liver and the values of the calculated parameters. (DOCX 1151 kb)

## Data Availability

The calculated values that used to support the findings of this study are included within the supplementary file. The raw (DICOM) data used to support the findings of this study are available from the corresponding author upon request.
