# Supplementary material for: Radiomic detection of microscopic tumorous lesions in small animal liver SPECT imaging
Source: EJNMMI Res. 2019 Jul 25;9:67. doi: 10.1186/s13550-019-0532-7 (PMC6658620; doi:10.1186/s13550-019-0532-7)
Supplement: Supplementary file 1 — The supplementary file contains the MATLAB script for calculating the parameters, additional example projections in each animal of the segmented liver and the values of the calculated parameters. (DOCX 1151 kb) [file 13550_2019_532_MOESM1_ESM.docx]

**Supplementary materials**

**Detection of tumorous lesions in the liver by a new, semi-quantitative data analysing technique based on small animal SPECT imaging**

Dániel S. Veres^1^, Domokos Máthé^2^, Nikolett Hegedűs^1^, Ildikó Horváth^1^, Fanni J. Kiss^1^, Krisztián Szigeti^1^

^1^ Department of Biophysics and Radiation Biology, Semmelweis University, H-1094, Budapest, Hungary

^2^ CROmed Translational Research Centers, H-1047 Budapest, Hungary

1. *MATLAB script for calculating the parameters*

%reading dicom files

[FileNames FilePaths] = uigetfile('*.dcm','Select the Dicom-files','Multiselect','on');

s=whos('FileNames');

if s.class=='char'

FileNames2={FileNames};

d=1;

else

FileNames2=FileNames;

size_filename = size(FileNames2);

d=size_filename(1,2);

end

%creating result matrix

results = cell (1+d, 6);

results(1,1:12) = {'filename', 'activity','volume', 'mean', 'skew', 'kurt' };

%calculations

for i=1:d

string = FileNames2{i};

info = dicominfo([FilePaths string]);

%storing data in a matrix

data = dicomread(info);

data = double(data);

%weighting factor for real activity

sv = info.RescaleSlope;

%voxel size

vm = info.PixelSpacing(1)*info.PixelSpacing(2)*info.SliceThickness*-1;

%calculating parameters of the VOI

activity = sum(data(:))*sv; %total activity

average=mean(data(data>0))*sv; %mean activity

skew = skewness(data(data>0),0); %skewness

kurt = kurtosis(data(data>0),0)-3; %excess kurtosis

voika=size(data(data>0)); %number of voxels

vol_voi = voika(1)*vm; %volume of the VOI

%writing result table

results(i+1,1) = {string};

results(i+1,2) = {activity};

results(i+1,3) = {vol_voi};

results(i+1,4) = {average};

results(i+1,5) = {skew};

results(i+1,6) = {kurt};

end

%saving results to excel file

[FileNames FilePaths] = uiputfile('*.xls','Save the results');

xlswrite(strcat(FilePaths,FileNames),results);

2. *Additional example projections in each animal of the segmented liver*


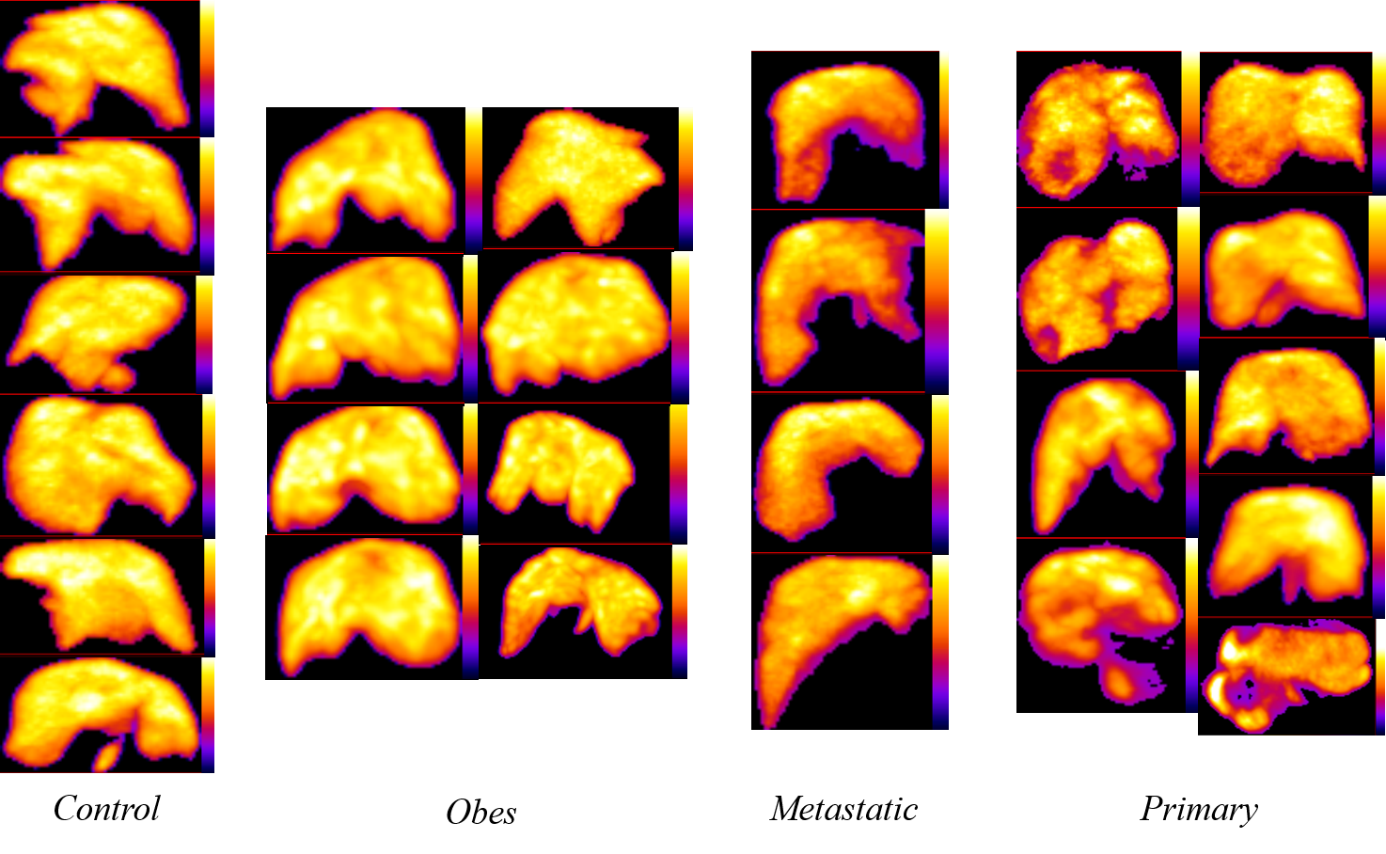


3. *Calculated parameters*

(O1 and O2: obserever 1 and observer 2)

| tumor | group | mass [g] | total activity [kBq] | liver activity O1 | volume [cm3] O1 | skewness O1 | kurtosis O1 | SUC O1 | SUV O1 |
| --- | --- | --- | --- | --- | --- | --- | --- | --- | --- |
| healthy | control | 23.1 | 83840 | 35048.8 | 1.29204 | -0.039867 | -1.14951 | 0.324 | 7.474 |
| healthy | control | 25.5 | 94380 | 50436.3 | 1.49558 | -0.060235 | -1.15189 | 0.357 | 9.112 |
| healthy | control | 42.8 | 101840 | 48382.1 | 1.3255 | -0.226858 | -1.05728 | 0.358 | 15.34 |
| healthy | control | 47.8 | 76920 | 33101.1 | 1.35349 | -0.229738 | -1.09275 | 0.318 | 15.198 |
| healthy | control | 31.6 | 98030 | 47429.5 | 1.20013 | -0.044094 | -1.14312 | 0.403 | 12.739 |
| healthy | control | 23.6 | 91200 | 44102.6 | 1.17805 | -0.004996 | -1.15449 | 0.41 | 9.688 |
| healthy | obes | 53.3 | 85770 | 42685.1 | 2.6054 | -0.247752 | -1.07616 | 0.191 | 10.181 |
| healthy | obes | 70.1 | 85790 | 40477 | 3.78988 | -0.059539 | -1.12174 | 0.124 | 8.727 |
| healthy | obes | 35.6 | 87030 | 39074.1 | 1.86099 | -0.115631 | -1.15568 | 0.241 | 8.589 |
| healthy | obes | 38.2 | 78770 | 37146.8 | 1.81174 | -0.184666 | -1.17989 | 0.26 | 9.943 |
| healthy | obes | 35.4 | 89090 | 40835.7 | 1.66603 | -0.144654 | -1.19865 | 0.275 | 9.739 |
| healthy | obes | 34 | 90410 | 36425.5 | 1.4447 | -0.106921 | -1.12864 | 0.279 | 9.482 |
| healthy | obes | 42.8 | 92930 | 41751.2 | 2.33103 | -0.308136 | -1.12202 | 0.193 | 8.249 |
| healthy | obes | 66 | 103110 | 46256.2 | 2.96594 | -0.308252 | -1.0203 | 0.151 | 9.983 |
| tumorous | primary | 36.2 | 102700 | 36267.9 | 2.9306 | 0.638962 | -0.12261 | 0.121 | 4.362 |
| tumorous | primary | 40.1 | 82240 | 36666.8 | 2.42323 | 0.383335 | -0.89729 | 0.184 | 7.378 |
| tumorous | primary | 36.8 | 76000 | 29292.2 | 1.96631 | 0.197054 | -1.05248 | 0.196 | 7.213 |
| tumorous | primary | 37.2 | 77590 | 35936.8 | 1.6719 | 0.088383 | -1.06023 | 0.277 | 10.305 |
| tumorous | primary | 38.9 | 76180 | 30455.4 | 1.54702 | 0.047519 | -1.1016 | 0.258 | 10.053 |
| tumorous | primary | 40.2 | 81700 | 29768.2 | 2.54088 | 0.433546 | -0.87084 | 0.143 | 5.765 |
| tumorous | primary | 36.6 | 77550 | 38430.4 | 1.8059 | 0.121035 | -0.98934 | 0.274 | 10.043 |
| tumorous | primary | 38.4 | 91480 | 26628.8 | 2.26563 | 0.559016 | -0.4846 | 0.128 | 4.934 |
| tumorous | primary | 42 | 79970 | 28438.4 | 5.14856 | 0.830803 | 0.679631 | 0.069 | 2.901 |
| tumorous | metastatic | 20.6 | 87240 | 38448 | 1.46977 | 0.44635 | -0.66484 | 0.3 | 6.177 |
| tumorous | metastatic | 22.2 | 88420 | 43791.8 | 1.7421 | 0.086349 | -0.92105 | 0.284 | 6.311 |
| tumorous | metastatic | 22 | 94310 | 45071.4 | 1.52944 | 0.130287 | -0.99153 | 0.312 | 6.874 |
| tumorous | metastatic | 20.4 | 81800 | 35620 | 1.51855 | 0.339948 | -0.809 | 0.287 | 5.85 |
|  |  |  |  | liver activity O2 | volume [cm3] O2 | skewness O2 | kurtosis O2 | SUC O2 | SUV O2 |
| healthy | control | 23.1 | 83840 | 34745.6 | 1.27136 | -0.04289 | -1.13996 | 0.326 | 7.53 |
| healthy | control | 25.5 | 94380 | 50436.3 | 1.49558 | -0.060235 | -1.15189 | 0.357 | 9.112 |
| healthy | control | 42.8 | 101840 | 48748.2 | 1.34403 | -0.226194 | -1.0698 | 0.356 | 15.243 |
| healthy | control | 47.8 | 76920 | 33101.1 | 1.35349 | -0.229738 | -1.09275 | 0.318 | 15.198 |
| healthy | control | 31.6 | 98030 | 47429.5 | 1.20013 | -0.044094 | -1.14312 | 0.403 | 12.739 |
| healthy | control | 23.6 | 91200 | 43904.7 | 1.16845 | -0.006896 | -1.15021 | 0.412 | 9.723 |
| healthy | obes | 53.3 | 85770 | 42685.4 | 2.60544 | -0.247751 | -1.07617 | 0.191 | 10.181 |
| healthy | obes | 70.1 | 85790 | 40477.7 | 3.78984 | -0.059522 | -1.1217 | 0.124 | 8.727 |
| healthy | obes | 35.6 | 87030 | 39077.8 | 1.86133 | -0.115584 | -1.15581 | 0.241 | 8.588 |
| healthy | obes | 38.2 | 78770 | 37146.8 | 1.81174 | -0.184666 | -1.17989 | 0.26 | 9.943 |
| healthy | obes | 35.4 | 89090 | 40513 | 1.64044 | -0.149411 | -1.18917 | 0.277 | 9.813 |
| healthy | obes | 34 | 90410 | 36425.5 | 1.4447 | -0.106921 | -1.12864 | 0.279 | 9.482 |
| healthy | obes | 42.8 | 92930 | 41299.6 | 2.28536 | -0.315824 | -1.10332 | 0.194 | 8.323 |
| healthy | obes | 66 | 103110 | 46581 | 3.0036 | -0.308691 | -1.03088 | 0.15 | 9.927 |
| tumorous | primary | 36.2 | 102700 | 35758.7 | 2.87421 | 0.637705 | -0.03434 | 0.121 | 4.385 |
| tumorous | primary | 40.1 | 82240 | 35985.2 | 2.33919 | 0.383282 | -0.89765 | 0.187 | 7.501 |
| tumorous | primary | 36.8 | 76000 | 28667.5 | 1.89069 | 0.191735 | -1.04004 | 0.2 | 7.342 |
| tumorous | primary | 37.2 | 77590 | 35242.9 | 1.61286 | 0.08894 | -1.04215 | 0.282 | 10.476 |
| tumorous | primary | 38.9 | 76180 | 30165.1 | 1.5205 | 0.045204 | -1.0917 | 0.26 | 10.13 |
| tumorous | primary | 40.2 | 81700 | 29768.2 | 2.54088 | 0.433546 | -0.87084 | 0.143 | 5.765 |
| tumorous | primary | 36.6 | 77550 | 38471.8 | 1.80941 | 0.120279 | -0.98982 | 0.274 | 10.035 |
| tumorous | primary | 38.4 | 91480 | 25184.7 | 2.05224 | 0.576822 | -0.44501 | 0.134 | 5.151 |
| tumorous | primary | 42 | 79970 | 27611.9 | 4.88783 | 0.830502 | 0.764545 | 0.071 | 2.967 |
| tumorous | metastatic | 20.6 | 87240 | 38430.8 | 1.46859 | 0.446095 | -0.66447 | 0.3 | 6.179 |
| tumorous | metastatic | 22.2 | 88420 | 43422.6 | 1.71481 | 0.091511 | -0.90777 | 0.286 | 6.358 |
| tumorous | metastatic | 22 | 94310 | 45169.3 | 1.5345 | 0.13418 | -0.9937 | 0.312 | 6.867 |
| tumorous | metastatic | 20.4 | 81800 | 35620 | 1.51855 | 0.339948 | -0.809 | 0.287 | 5.85 |
